# Diagnostic imaging capabilities of the Ocelot -Optical Coherence Tomography System, ex-vivo evaluation and clinical relevance

**DOI:** 10.1186/s12880-015-0098-4

**Published:** 2015-11-18

**Authors:** Suhail Dohad, John Shao, Ian Cawich, Manish Kankaria, Arjun Desai

**Affiliations:** Cardiovascular Medical Group of Southern California, Beverly Hills, CA USA; Cedars Sinai Heart Institute, Cedars Sinai Medical Center, Los Angeles, CA USA; Columbia University Hospital, New York, NY USA; Arkansas Heart Hospital, Little Rock, AK USA; Avinger, Inc, Redwood City, CA USA

## Abstract

**Background:**

Optical coherence tomography (OCT) is a high-resolution sub-surface imaging modality using near-infrared light to provide accurate and high contrast intra-vascular images. This enables accurate assessment of diseased arteries before and after intravascular intervention.

This study was designed to corroborate diagnostic imaging equivalence between the Ocelot and the Dragonfly OCT systems with regards to the intravascular features that are most important in clinical management of patients with atherosclerotic vascular disease. These intravascular features were then corroborated in vivo during treatment of peripheral arterial disease (PAD) pathology using the Ocelot catheter.

**Methods:**

In order to compare the diagnostic information obtained by Ocelot (Avinger Inc., Redwood City, CA) and Dragonfly (St. Jude Medical, Minneapolis, MN) OCT systems, we utilized ex-vivo preparations of arterial segments. Ocelot and Dragonfly catheters were inserted into identical cadaveric femoral peripheral arteries for image acquisition and interpretation.

Three independent physician interpreters assessed the images to establish accuracy and sensitivity of the diagnostic information. Histologic evaluation of the corresponding arterial segments provided the gold standard for image interpretation.

In vivo clinical images were obtained during therapeutic interventions that included crossing of peripheral chronic total occlusions (CTOs) using the Ocelot catheter.

**Results:**

Strong concordance was demonstrated when matching image characteristics between both OCT systems and histology. The Dragonfly and Ocelot system’s vessel features were interpreted with high sensitivity (91.1–100 %) and specificity (86.7–100 %). Inter-observer concordance was documented with excellent correlation across all vessel features. The clinical benefit that the Ocelot OCT system provided was demonstrated by comparable procedural images acquired at the point of therapy.

**Conclusions:**

The study demonstrates equivalence of image acquisition and consistent physician interpretation of images acquired by the Ocelot and the Dragonfly OCT systems in-spite of distinct image processing algorithms and catheter configurations. This represents a dramatic shift away from both fluoroscopic imaging and diagnostic-only OCT imaging during peripheral arterial intervention towards therapeutic devices that incorporate real time diagnostic OCT imaging. In the clinical practice, these diagnostic capabilities have translated to best-in-class safety and efficacy for CTO crossing using the Ocelot catheter.

## Background

The ability to have accurate and catheter based intravascular imaging provides interventional physicians with invaluable insight into the procedural vascular anatomy [1]. Luminal angiography has long been the gold standard for visualizing the arterial lumen and is routinely used for arterial reconstruction. Nevertheless, the structural composition of diseased arterial beds is not adequately visualized by angiography [2]. Intravascular imaging provides important structural information, which is frequently used for diagnostic and therapeutic decision making during the interventional procedure. The highest resolution source for intravascular imaging, optical coherence tomography (OCT), is a sub-surface imaging modality using near-infrared light [3].

Physicians use OCT to evaluate, characterize and understand intravascular anatomic features of normal and abnormal vessels. OCT accomplishes this via emission of a light source and no additional ioninizig radiation.

Initially, intravascular ultrasound (IVUS) provided a solution for in-vivo arterial assessment of luminal dimensions, plaque distribution and morphology, aneurysmal disease [4, 5], vulnerable plaque with and without rupture [6] and stent mal-apposition [7]. Based on the same physical principles of IVUS, OCT substitutes light waves for sound waves and provides a substantial increase in resolution as compared to IVUS and high definition IVUS [8]. This directly translates to a clinical benefit where microscopic disease processes can be evaluated both before and after an endovascular intervention [9]. For example, physicians are able to appreciate thin-capped fibro-atheromas [10], vascular healing following stent deployment [11, 12] and the extent of stent mal-apposition not reliably identifiable using IVUS [13]. A significant amount of research has validated OCT’s histologic equivalence and ability to determine reference vessel diameter, minimal luminal diameter, lesion length and composition (calcium, lipid, thin cap, etc.) [14–16]^.^ Accordingly, the international working group on intravascular optical coherence tomography (IWG-IVOCT) recently published consensus standards defining terminology for the interpretation of intra vascular OCT (IVOCT) [17].

The objective of this study was to illustrate the diagnostic capabilities of the Ocelot -OCT system. In order to validate the imaging capabilities of the Ocelot, we performed a comparative evaluation of the Ocelot and the Dragonfly OCT systems. The Ocelot and Dragonfly OCT systems individually evaluated identical intravascular features that are most important in clinical management of atherosclerotic vascular disease (as described in details in the Material and Methods section).

While several studies [15–18] have compared independent OCT system images with histology for technical validation, this is the first study comparing OCT images from two different systems to both histology and physician interpretation. Subsequently, and to further illustrate the diagnostic capabilities of the Ocelot -OCT system, we present a series of representative in-vivo images, that were acquired by the Ocelot OCT system during interventions. These images match the features that were evaluated ex-vivo, including bifurcation, calcification, stent struts, and thrombus.

## Methods

### OCT devices description

The Ocelot system is designed to cross chronic total occlusions (CTOs) facilitated by OCT in the peripheral vasculature. The Dragonfly System is intended for OCT guided diagnostic imaging of the coronary arteries. Both imaging consoles use swept-source optical coherence tomography and obtain a radial tomograph of the artery by acquiring individual A-lines as the optical beam is rotated along the axis of the catheter inside the artery. Table 1 outlines the comparison for the basic operational features of the Ocelot and the Dragonfly devices. The two systems differ with regards to the A-line acquisition modes, the positioning of the optical fiber on the catheter, the catheter rotation speed, the optical fiber rotation speed, and the modes of OCT image display.

**Table 1:** Ocelot and Dragonfly OCT system technical specifications

Technical specifications	Dragonfly system	Ocelot system
Laser	Swept Source	Swept Source
Domain	Frequency	Frequency
Interferometer	Differential Path	Common Path
Imaging Speed	100 Hz	1.0 Hz
Laser Scan Rate	50,000 axial lines/s	20,000 axial lines/s
Sensitivity	>90 dB	>90 dB
Dynamic Range	>50 dB	>50 dB
Imaging Range	≤5 mm	≤3.3 mm
Resolution	<20 μm (axial) 25-60 μm (lateral)	<20 μm (axial) < 300 μm (lateral)
Tissue Penetration	1–2 mm	1–2 mm

### Tissue preparation

**Fig. 1:**
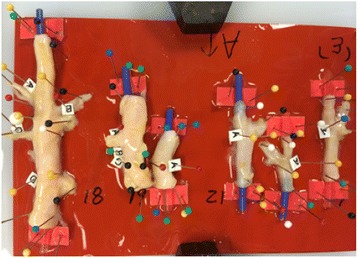
Human cadaveric vessel segments that were utilized to compare the diagnostic information obtained by the Ocelot and Dragonfly OCT systems

In order to compare the diagnostic information obtained by the Ocelot and Dragonfly OCT systems we utilized human cadaveric vessels: the arterial segments were excised from the superficial femoral artery to the peroneal and sectioned into individually labeled segments (Fig. 1).

In order to eliminate the need for blood dispersion and enable the ex-vivo arterial imaging, the cadaveric arterial segments were held fixed in saline bath solutions.

The cadaveric arteries were procured from: Science Care, 21210 N19th Ave. Phoenix, AZ 85027. All donors (or their next of kin) consented in writing, and all the written consents are on file with Science Care.

The donor procurement was in compliment with the Declaration of Helsinki and the local research ethics committee: Medical Affairs at Avinger, approved the donation protocol as well as the study utilization.

### Device tissue imaging

**Fig. 2:**
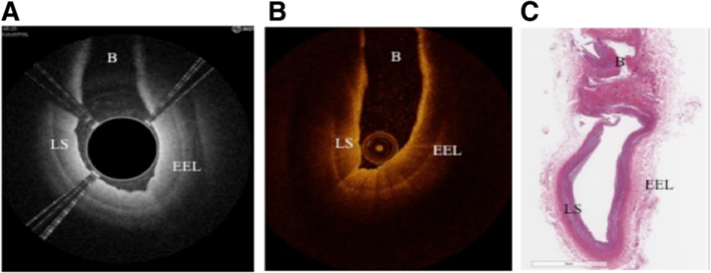
Arterial segments, ex-vivo, depicting Layered Structure (LS), Bifurcation (B), and External Elastic Lamina (EEL) as seen in Ocelot (left), Dragonfly (center) and histology (right)

**Fig. 3:**
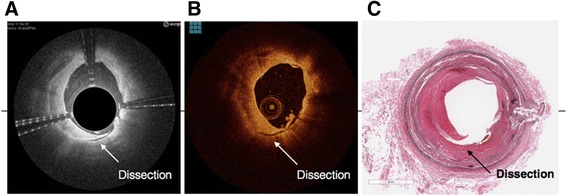
Ex-vivo segment with Dissection (D) seen in Ocelot (left), Dragonfly (center) and histology (right)

**Fig. 4:**
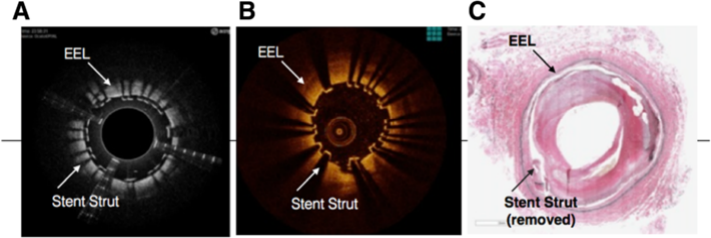
Stented cadaveric arterial segment, Non-Layered Structure (NLS) and External Elastic Lamina (EEL) as seen in Dragonfly (left), Ocelot (center) and histology (right) images

**Fig. 5:**
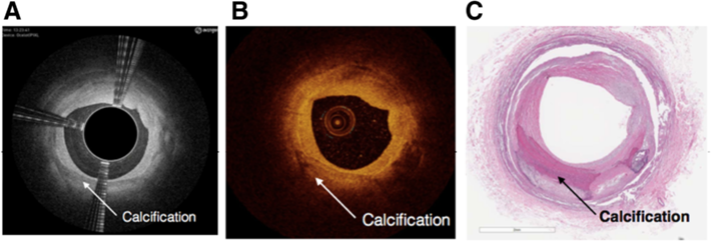
Ex-vivo segment with Non Layered Structures (Calcification)

**Fig. 6:**
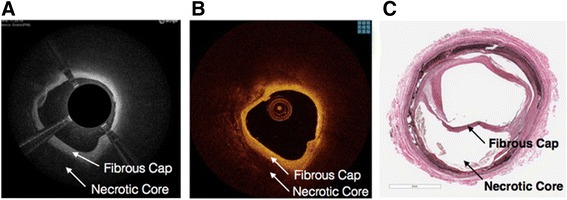
Ex-vivo segment with Non Layered Structures (Necrotic Core with Fibrous Cap)

Both the Ocelot and Dragonfly catheters were inserted into identical vessel segments for image acquisition and comparison. The image acquisition was performed according to standard operational procedures respective for each device. The cadaveric vessel segment subsets that were used for comparative image analysis were chosen based on screening for the following features (further described in Table 2): intact vessel-wall architecture of intima, media and adventitia in a single segment (defined as Layered Structure, Table 2) well preserved external elastic lamina (EEL), arterial segment with bifurcation, dissection, stent, or demonstrating arterial disease, including calcium, fibrin, lipid, thrombus, or a combination thereof. Table 2 also outlines the nomenclature that was used to standardize the OCT imaging evaluation. Accordingly, a total of 52 image sets (from *n* = 9 donors and 36 vessels) were selected and OCT images were acquired with the Ocelot and Dragonfly catheters. Figs. 2, 3, 4, 5 and 6 depict a representative images obtained with the Ocelot OCT system (Left panel -A), images obtained with the Dragonfly OCT system (Middle panel -B), and the matching histology slides (Right panel -C). Figure 2 shows representative images of the following features: Layered Structures (LS), e.g. the vessel-wall architecture of intima, media and adventitia (LS, Fig. 2), arterial Bifurcation or arterial Branch (b, Fig. 2) and the External Elastic Lamina (EEL, Fig. 2). Concordantly, Fig. 3 shows representative OCT image of an arterial dissection as captured by the Ocelot OCT system (Fig. 3, left panel -a), by the Dragonfly OCT system (Fig. 3, middle panel -b) and the matching histology slide (Fig. 3, right panel -c). Figure 4 shows representative imaging of a stented artery segment as captured by Ocelot (Fig. 4, left panel -a), or by Dragonfly OCT (Fig. 4, middle panel -b) systems. The matching histological slide (Fig. 4, right panel -c) shows representative slide of a stented arterial segment, notice the histological processing necessitated the removal of the metallic stent from the fixed artery. Figure 5 shows representative image of calcification in the arterial segment as captured by the Ocelot OCT system (Fig. 5, left panel -a), by the Dragonfly OCT system (Fig. 5, middle panel -b) and the matching histology slide (Fig. 5, right panel -c). Representative atherosclerotic plaque in the arterial segment is shown in Fig. 6. The boundary of the fibrous cap encapsulating the necrotic core and the distortion of the normal vessel-wall architecture are apparent from the representative histological slide (Fig. 6, left panel –c).

**Table 2:** Nomenclature for OCT evaluated features

Nomenclature	Description
Layered Structure (LS)	Layered structure images define the presence of the normal vessel wall architecture of intima, media and adventitia, which is characterized by a layered architecture varying in contrast to each other and may appear as (1) a thin bright signal intima (not always discernible), (2) a dark signal media, and (3) a bright and heterogeneous signal adventitia is a sample image set showing layered structure.
Bifurcation (B)	The point at which one arterial lumen communicates with a separate arterial lumen, which is characterized as a luminal space with contiguous borders between communicating lumens is a sample image set showing bifurcation.
Dissection (D)	A disruption of the vessel wall classified as any or all of the following: intima, media, adventitia, intramural hematoma or intra-stent and characterized by (1) the presence of a false lumen and/or (2) identification of a tissue flap.
Stent	The presence of an implanted material in the vessel structure, which is characterized by a distinct line followed by an absence of imaging of deeper vessel structures
Non-Layered Structure (NLS)	The presence of disease in the vessel structure, which is characterized by (1) a loss of a layered structure and/or (2) localized thickening of a layer. This may include calcium, fibrin, lipid, thrombus, or a combination thereof.
External Elastic Lamina (EEL)	The presence of the border between the media and adventitia, which is characterized as a contrasting border between the two outermost layers of the vessel structure.

Representative images of these features are shown respectively, as captured by the Ocelot OCT imaging system (Fig. 6, left panel-a) or the Dragonfly OCT system (Fig. 6, middle panel-b).

**Fig. 7:**
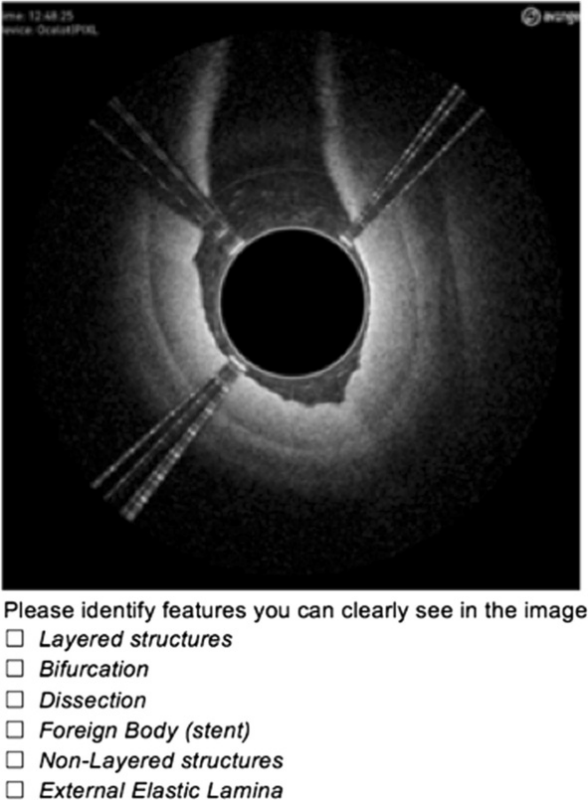
Sample Test Question (Ocelot)

**Fig. 8:**
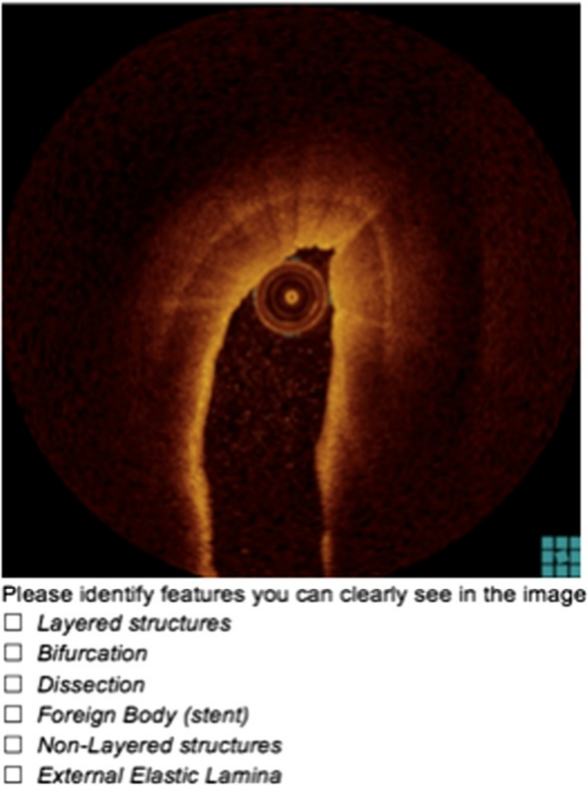
Sample Test Question (Dragonfly)

Figures 7 and 8 demonstrate how physician interpreters were presented the image and answer options for a matched image set. These images appeared independently and were randomly ordered within the full test cohort.

### Study design

The cadaveric study was designed to evaluate the concordance between the Ocelot and the Dragonfly OCT systems.

Three independent physician interpreters with significant experience using both Dragonfly and Ocelot OCT systems (Table 3) evaluated the images for the presence of each feature, based on their clinical understanding of OCT and the published literature [15, 18].

**Table 3:** Independent Physician Interpreter OCT Experience

Physician interpreter	Dragonfly experience (months)	Ocelot experience (months)
1	26	21
2	33	4
3	31	8

In order to prevent any bias and or comparison amongst image sets, all images were presented in a random fashion within each system, meaning the image sets were not shown together. Each physician interpreter was blinded to a unique sequence of the images. During the physicians review process, an electronic survey (Survey Gizmo, Boulder, CO, USA) captured all answer sets.

The clinical images were obtained during therapeutic interventions that included CTO crossing using the Ocelot catheter. The diagnostic information was obtained after an informed consent.

### Histological analysis

Third party histologic evaluation (Pathology Research Laboratories, South San Francisco, CA) was performed using core lab standard rating scales.

### Statistical analysis

Statistical boundaries for image comparison were set in concordance with diagnostic imaging standards. Tolerating a less than or equal to −20.0 % difference in sensitivity (positive features identification) or specificity (false positive features identification) for imaging performance between Ocelot and Dragonfly was deemed acceptable for an individual arterial feature. The composite across all arterial features was tightened to less than or equal to −15.0 % to match OCT vessel features with verified histology, establishing a minimum of 85 % accuracy for both Ocelot and Dragonfly.

Sample size determination was based on the binomial distribution of one-sided 95 % lower confidence boundary for the sensitivity difference between Ocelot and Dragonfly with a minimum number of matched sets providing a high probability (80 %) of success with a specified assessment compared to histology.

The calculated lower bound required a minimum of 15 matched image sets, one Ocelot and one Dragonfly fly image, per arterial feature. A total sample size of 52-matched image sets was used to meet the requisite individual and composite feature requirements or analysis.

Following blinded physician analysis, kappa statistics were calculated to determine the significance of inter-observer variability across arterial feature identification.

## Results and Discussion

 The three independent physician interpreters demonstrated strong concordance when matching image characteristics between both OCT systems and histology. Table 3 highlights the three-way analysis used to categorize physician interpretation of images where a vessel feature is or is not present in the 1) Ocelot image, 2) Dragonfly image, and 3) Histologic sample. The interpretation of each vessel feature was independently analyzed across all images reviewed.

Across the six image components tested, the layered structures (intima, media, and adventitia), the stent struts and the arterial bifurcation had the lowest levels of discordance measured amongst physician interpreters. These three individual components were interpreted with a 0.0 % difference between Dragonfly and Ocelot. This correlation is consistent with the “high” level of evidence describing these three morphologies in the international working group for intravascular OCT (IWG-IVOCT) consensus standards [17].

Non-layered structures (atheromatous disease) and external elastic lamina (EEL) had 1.1 % and 1.0 % levels of discordance respectively. While atheromatous disease and its components (fibrous, fibro calcific, necrotic core, etc.) are represented by high levels of evidence in the IWG-IVOCT consensus standards, deep wall borders of the artery and EEL are less well defined. Regardless, the matched sensitivity of these comparative images is sufficiently high to determine diagnostic interpretative equivalence.

In addition to individual vessel feature identification, a kappa statistic was calculated to determine the inter-observer agreement (Table 4). Kappa values measured above 0.8 are considered statistically strong correlates [19]. There was a significant consistency observed between all physician interpreters with kappa values ranging from 0.82 to 1.0 across all vessel features and between the two OCT platforms.

**Table 4:** Comparative Statistics for Ocelot and Dragonfly OCT systems

Feature	OCT system	Sensitivity	Diagnosed	Kappa statistic
Layered Structures	Ocelot	96.3	93.3	0.90
	Dragonfly	96.3	92.0	0.88
Non-Layered Structures	Ocelot	97.8	92.1	0.91
	Dragonfly	96.8	96.8	0.93
Bifurcation	Ocelot	100.0	100.0	1.0
	Dragonfly	100.0	100.0	1.0
Dissection	Ocelot	91.0	100.0	0.93
	Dragonfly	93.3	100.0	0.95
EEL	Ocelot	95.8	93.3	0.89
	Dragonfly	94.8	86.7	0.82
Stent	Ocelot	100.0	100.0	1.0
	Dragonfly	100.0	100.0	1.0

In Summary, the Dragonfly and Ocelot OCT system’s vessel features were interpreted with an overall high sensitivity (91.1–100 %) and specificity (86.7–100 %). There were no differences in sensitivity between Ocelot and Dragonfly by physician of more than 7.4 %; and similarly, there were no differences in specificity between Ocelot and Dragonfly of more than 10 %. These endpoint values are consistent with diagnostic equivalence, falling well below the statistical upper limit of −20 % sensitivity and −15 % specificity difference level.

The matched sensitivities and specificities suggest that both OCT systems accurately characterize the true histology of peripheral arterial disease. Additionally, the high level of concordance demonstrates consistent clinical interpretations of the images. The improved resolution of OCT when compared with IVUS may explain the consistent and accurate image interpretation between physicians and across all vessel features.

With diagnostic equivalence to Dragonfly (current FDA approved OCT imaging modality), interventional physicians can reliably use real time Ocelot images to understand vascular structures while guiding therapeutic intervention across arterial chronic total occlusions [19]. This is inherently different from stand-alone diagnostic OCT catheters, like Dragonfly, which are limited to imaging only without any simultaneous therapeutic applications. The ability for one catheter to combine both diagnostic OCT with therapeutic OCT guidance may provide several benefits to physicians and patients. These benefits include increased clinical safety and efficacy, procedural time reduction (see while treating instead of see then treat), reduced healthcare consumption (two devices in one), and reduced radiation exposure.

**Fig. 9:**
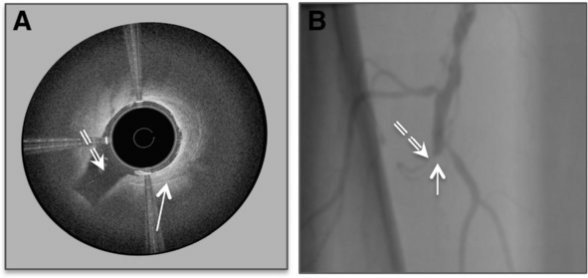
Arterial branch (annotated by the dashed arrow) detected by the Ocelot OCT catheter in the course of CTO recanalization (panel **a**) and the corresponding angiographic image (panel **b**). The strait arrow indicates chronic occlusion due to a fibrotic plaque

**Fig. 10:**
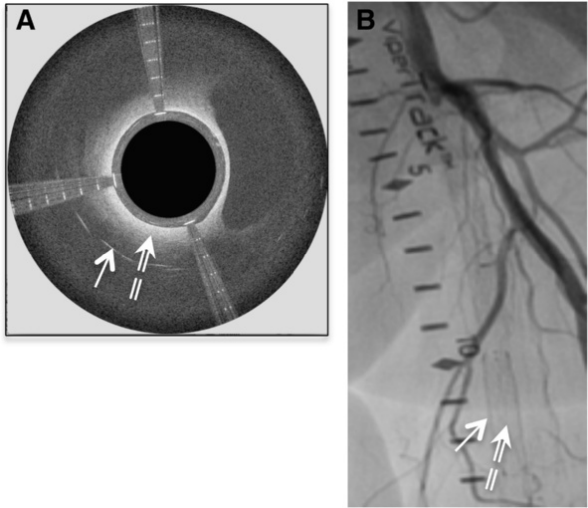
In stent restenosis depicted by the Ocelot OCT catheter in the course of CTO recanalization (panel **a**) and the corresponding angiographic image (panel **b**). The strait arrow annotates the stent struts, and the dashed arrow annotates the occlusive neo-intimal tissue

**Fig. 11:**
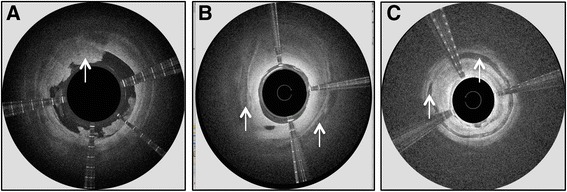
Arterial features detected by the Ocelot OCT system during CTO recanalization (indicated by an arrow in the image); (**a**) thrombotic burden in the occluded artery, (**b**) calcium nodules embedded in the adventitia, and (**c**) calcium aggregates in the medial arterial layer

Figures 9, 10 and 11 demonstrate the direct clinical benefit provided by including diagnostic imaging at the point of therapy in the Ocelot platform technology. Procedural images acquired by the Ocelot OCT system during clinical interventions as well as their respective procedural angiographic images (Figs. 9 and 10) highlight how the diagnostic OCT image provided direct therapeutic guidance not available with conventional angiography or stand alone diagnostic OCT.

Figure 9 demonstrates an arterial branch as depicted by the Ocelot OCT catheter and the corresponding angiographic image. The OCT guidance that Ocelot provided during this intervention enabled clear intravascular visualization of the bifurcating branch during the CTO crossing, eliminating unnecessary collateral tracking or potential injury.

Figure 10 shows detection of stent struts in the occluded in-stent restenosis artery and the corresponding angiographic image. OCT guidance enabled intra-stent crossing with real-time confirmation of catheter positioning. Figure 11 depicts additional arterial features that were detected by the Ocelot OCT system and impacted the subsequent therapeutic algorithms; A. thrombotic burden in the occluded artery, B. calcium nodules embedded in the adventitia, and C. calcium aggregates in the medial arterial layer.

Future applications combining real time OCT guidance during therapeutic intervention may further serve to improve outcomes and procedural efficiencies when treating both peripheral and coronary arterial disease. Such technologies may include OCT guided directional atherectomy, true lumen re-entry, stent deployment systems, and local drug delivery mechanisms.

Limitations of this study include comparative testing within an ex-vivo in (bloodless) environment and physician image interpretation using single frame images, as opposed to live OCT files. Additionally, future studies should consider including intra-observer variance measurement. As a result of strong inter-rater agreement measured in this study, intra-rater assessment was not pursued.

## Conclusions

The comparison of Ocelot and Dragonfly via the cadaverous model successfully validated comparable identification the most pertinent vascular anatomies and pathologies, encountered during peripheral arterial interventions, including bifurcations, calcium or arterial dissections.

Accordingly, the diagnostic capabilities of the Ocelot OCT system demonstrated in this paper have been successfully translated into clinical best-in-class safety (98 %) and efficacy (97 %) for peripheral CTO crossing as demonstrated by the pivotal CONNECT II trial [19].

## Abbreviations

OCT: Optical coherence tomography
IWG-IVOCT: Intravascular optical coherence tomography
CTO: Chronic total occlusions
EEL: External elastic lamina
LS: Layered Structure
NLS: Non layered structures, SFA
